# A collection of crabs (Crustacea, Brachyura) from the southwestern coast of India, with a discussion of the systematic position of *Nectopanope* Wood-Mason in Wood-Mason & Alcock, 1891 (Euryplacidae)

**DOI:** 10.3897/zookeys.818.32108

**Published:** 2019-01-17

**Authors:** Peter K.L. Ng, P. Priyaja, A. Biju Kumar, S. Suvarna Devi

**Affiliations:** 1 Lee Kong Chian Natural History Museum, Faculty of Science, National University of Singapore, 2 Conservatory Drive, 117377, Singapore National University of Singapore Singapore Singapore; 2 Department of Marine Biology, Microbiology and Biochemistry, School of Marine Sciences, Cochin University of Science and Technology, Cochin - 682 016, Kerala, India Cochin University of Science and Technology Cochin India; 3 Department of Aquatic Biology and Fisheries, University of Kerala, Kariavattom, Thiruvananthapuram – 695581, Kerala, India University of Kerala Thiruvananthapuram India

**Keywords:** Brachyura, Euryplacidae, Indian Ocean, new records, rare species, revised taxonomy, systematics

## Abstract

A report on the brachyuran crabs collected from the southwestern coast of India by the Indian research vessel FORV *Sagar Sampada* is presented. The material consists of 13 species from three genera and five families, of which four are new records for India: *Heteroplaxmaldivensis* (Rathbun, 1902) (Euryplacidae), *Cryptopodiacollifer* Flipse, 1930 (Parthenopidae), *Thalamitamacrodonta* Borradaile, 1903 (Portunidae), and *Paraxanthodescumatodes* (MacGilchrist, 1905) (Xanthidae). The cruise also obtained the first known male of *Nectopanoperhodobaphes* Wood-Mason in Wood-Mason & Alcock, 1891 (type species of *Nectopanope* Wood-Mason in Wood-Mason & Alcock, 1891), and its characters show that it is in fact a member of the Euryplacidae Stimpson, 1871. The genus had previously been incorrectly classified in the Xanthidae MacLeay, 1838.

## Introduction

We here report on a small but noteworthy collection of brachyuran crabs obtained by a fishery research vessel off the southwestern coast of India in 2017. While consisting of only 13 species from three genera and five families, the material obtained includes several rare species, including one which has not been seen since 1891.

The discovery of a male of *Nectopanoperhodobaphes* Wood-Mason in Wood-Mason & Alcock, 1891, is significant as the family position of the genus *Nectopanope* Wood-Mason in Wood-Mason & Alcock, 1891, has been uncertain, because it was previously known only from the type female. The male characters show that *Nectopanope* is a member of Euryplacidae Stimpson, 1871, and close to *Psopheticoides* Sakai, 1969, from the western Pacific. *Nectopanope* is rediagnosed, while *Nectopanoperhodobaphes* is redescribed and figured. A male of the rarely reported parthenopid *Cryptopodiacollifer* Flipse, 1930, not previously known from India, is figured. The euryplacid *Heteroplaxmaldivensis* (Rathbun, 1902), the rarely reported portunid *Thalamitamacrodonta* Borradaile, 1903, and the xanthid *Paraxanthodescumatodes* (MacGilchrist, 1905), are also recorded from India for the first time.

## Materials and methods

All specimens were collected during an exploratory survey (cruise 360) of FORV *Sagar Sampada* belonging to the Center for Marine Living Resources & Ecology (CMLRE) under the Ministry of Earth Sciences, India, in May 2017, conducted along the southwestern coast of India. Specimens were collected using grabs and dredged from depths ranging from 50–200 m. The material studied is in the museum collections of the Department of Aquatic Biology and Fisheries, University of Kerala (DABFUK).

Measurements provided are of the maximum carapace width and length, respectively. The classification and terminology used follows Ng et al. (2008) and Davie et al. (2015a, b). Complete synonymies are only provided for species which are treated at length.

## Systematics

### Family Raninidae De Haan, 1839

#### *Notosceles* Bourne, 1922

##### Notosceles serratifrons

Taxon classificationAnimaliaDecapodaRaninidae

(Henderson, 1893)

Fig. 1A

###### Material examined.

2 males (9.1 × 17.6 mm, 9.0 × 17.5 mm), 8°19.972'N, 76°35.897'E, 100 m.

###### Remarks.

Henderson (1893) described this species from Sri Lanka. It has since been found in India (Alcock 1896; Dev Roy 2013; Trivedi et al. 2018) as well as Australia, Japan, mainland China and Taiwan (Sakai 1976; Chen and Sun 2002; Ahyong et al. 2009).

**Figure 1.**
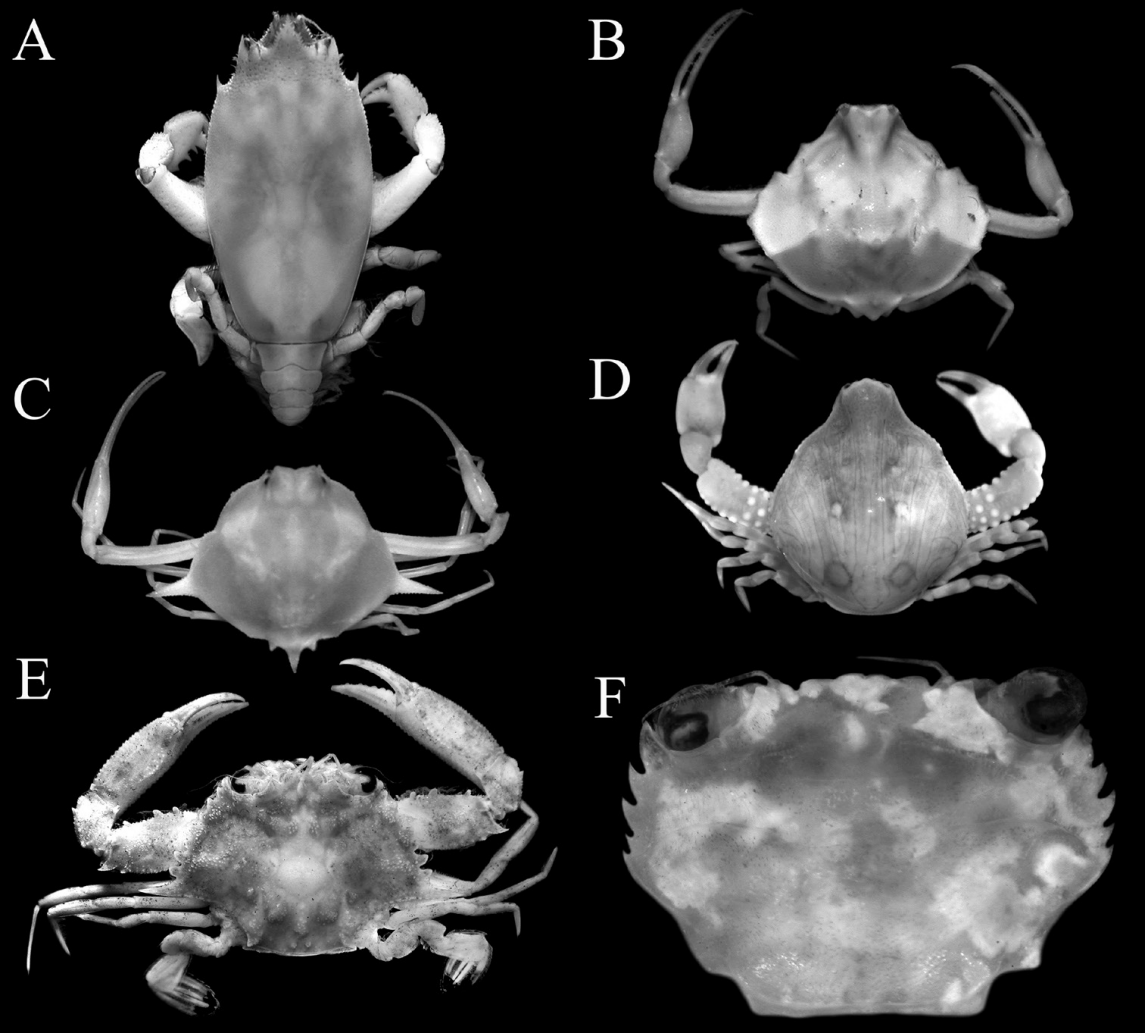
Overall dorsal habitus. **A***Notoscelesserratifrons* (Henderson, 1893), male (9.1 × 17.6 mm) **B***Nursiliatonsor* Alcock, 1896, female (6.0 × 5.3 mm) **C***Arcaniagracilis* Henderson, 1893, male (7.7 × 5.5 mm) **D***Coleusiaurania* (Herbst, 1801), female (10.1 × 12.0 mm) **E***Xiphonectestuberculosus* (A. Milne-Edwards, 1861), male (21.1 × 12.4 mm) **F***Thalamitamacrodonta* Borradaile, 1902, female (8.4 × 5.8 mm).

### Family Leucosiidae Samouelle, 1819

#### *Nursilia* Bell, 1855

##### Nursilia tonsor

Taxon classificationAnimaliaDecapodaLeucosiidae

Alcock, 1896

Fig. 1B

###### Material examined.

1 young female (6.0 × 5.3 mm), 8°22.727'N, 76°43.545'E, 50 m.

###### Remarks.

The species was first described from the Andaman Sea (Alcock 1896) and has since been reported from other parts of India, Southeast Asia, China, and Japan (Sakai 1976; Serène and Soh 1976; Tan 1996; Chen and Sun 2002; Dev Roy and Nandi 2012).

#### *Arcania* Leach, 1817

##### Arcania gracilis

Taxon classificationAnimaliaDecapodaLeucosiidae

Henderson, 1893

Fig. 1C

###### Material examined.

1 juvenile male (7.7 × 5.5 mm), 7°16.713'N, 77°37.582'E, 200 m.

###### Remarks.

The genus was revised by Galil (2001) who confirmed that the two Indian Ocean species, *Arcaniaquinquespinosa* Alcock & Anderson, 1894, and *A.gracilis* Henderson, 1893, are subjective synonyms. The species has a wide distribution in India and the Indo-West Pacific (see Galil 2001; Trivedi et al. 2018).

#### *Coleusia* Galil, 2006

##### Coleusia urania

Taxon classificationAnimaliaDecapodaLeucosiidae

(Herbst, 1801)

Fig. 1D

###### Material examined.

1 juvenile female (10.1 × 12.0 mm), 7°27.978'N, 77°32.297'E, 100 m.

###### Remarks.

The identity of this species and the confused status of the types were resolved by Ng et al. (2014). The species has a wide range in the Indo-West Pacific (see also Ng et al. 2014; Promdam et al. 2014).

### Family Portunidae Rafinesque, 1815

#### *Xiphonectes* A. Milne-Edwards, 1873

##### Xiphonectes tuberculosus

Taxon classificationAnimaliaDecapodaPortunidae

(A. Milne-Edwards, 1861)

Fig. 1E

###### Material examined.

1 male (21.1 × 12.4 mm), 1 female (18.6 × 10.1 mm), 7°27.978'N, 77°32.297'E, 200 m.

###### Remarks.

This species was described from Hawaii but has since been reported from all across the Indo-West Pacific to Madagascar (A. Milne-Edwards 1861; Stephenson 1972a; Davie 1987). In India, it has previously been reported from Tamil Nadu and the Andaman Sea (Alcock 1894, 1899b; Dev Roy 2015; Dev Roy and Nandi 2007, 2012).

#### *Monomia* Gistel, 1848

##### Monomia argentata argentata

Taxon classificationAnimaliaDecapodaPortunidae

(A. Milne-Edwards, 1861)

###### Material examined.

1 young male (18.2 × 10.2 mm), 8°58.270'N, 76°17.365'E, 50 m.

###### Remarks.

This is a well-known and widely distributed species in the Indo-West Pacific (Stephenson 1972b; Apel and Spiridonov 1998); and is found in most states in India (Trivedi et al. 2018).

#### *Thalamita* Latreille, 1829

##### Thalamita macrodonta

Taxon classificationAnimaliaDecapodaPortunidae

Borradaile, 1902

Fig. 1F

###### Material examined.

1 young female (8.4 × 5.8 mm), 8°22.727'N, 76°43.545'E, 50 m.

###### Remarks.

Borradaile (1902) described *Thalamitaexetasticamacrodonta* from two specimens from two islands in the Maldives, Kolumadulu and Suvadiva. Crosnier (1975) examined the syntypes and commented that the two specimens are not conspecific. He noted that the specimen from Kolumadulu Island was almost certainly *T.sexlobata* Miers, 1886, while the other from Suvadiva Island is the actual *T.macrodonta* which he treated as a distinct species. Apel and Spiridonov (1998) re-examined the type material and selected the second syntype from Suvadiva as the lectotype of *T.macrodonta* s. str.

The present specimen from India is incomplete and not in good condition, but agrees with the description and figures of *T.macrodonta* by Crosnier (1975: fig. 4c, d) and Apel and Spiridonov (1998: fig. 59).

### Family Euryplacidae Stimpson, 1871

#### Nectopanope

Taxon classificationAnimaliaDecapodaEuryplacidae

Wood-Mason in Wood-Mason & Alcock, 1891

Nectopanope Anonymous, 1891: 56 (nomen nudum).
Nectopanope Wood-Mason in Wood in Wood-Mason & Alcock, 1891: 261.

##### Diagnosis.

Carapace (Fig. 3A, B) subhexagonal, wider than long, dorsal surface smooth, regions poorly indicated; front (Fig. 3C–E) wide, straight with small median notch, with transverse sulcus along margin. Anterolateral borders (Fig. 3A, B) convex; with two low teeth posterior to broadly triangular outer orbital angle, first tooth wider than acute second tooth. Orbits (Fig. 3C–E) wide, spherical, deep; supraorbital margin with submedian notch, small acute lobe before notch with front; low suborbital tooth on broad, suborbital border; eye peduncles short, stout, with large subreniform (dorsoventrally flattened) cornea (Fig. 36D, E). Basal antennal article mobile, completely closing orbital hiatus (Fig. 3D). Ischium of third maxilliped (Fig. 3B) elongate; anteroexternal margin of merus auriculiform. Cheliped fingers (Figs 3A, G, 4E, F) stout, as long as propodus, not pigmented; carpus with small, sharp spine on inner margin, merus with acute anterodorsal tooth. Dorsal margins of ambulatory legs (P2–P5) (Figs 3A, 4G–J) meri, carpi, propodi unarmed, dactyli slender, smooth, setose; P5 propodus, dactylus proportionally short, flattened, fringed with many short setae. Thoracic sternum (Fig. 4A, D) relatively wide; sternites 1, 2 completely fused; suture 2/3 complete, gently convex towards buccal cavity; sternites 3, 4 medially fused, with shallow median groove, almost indiscernible with only lateral notch distinct; sutures 4/5, 6/7, 7/8 medially interrupted, 5/6 complete; median groove on thoracic sternites 7, 8. Male sternopleonal cavity (Fig. 4A, D) deep, reaching median part of sternite 4, just before sternite 3. Press-button of male pleonal-locking mechanism (Fig. 4D) present as low tubercle on sternite 5, near thoracic suture 4/5. Male pleon (Fig. 4A–C) narrow, slender, T-shaped, lateral margins of somites 4–6 abruptly narrowing from somite 3 to transversely narrow, acutely triangular telson (Fig. 4B); somite 3 wide, reaching inner margins of P5 coxae; no part of thoracic sternite 8 exposed by closed pleon, somite 2 transversely shorter than somite 3, somite 1 (Fig. 4C) conspicuous, narrow. G1 (Fig. 7A–D) long, slender, almost straight; distal quarter distinctly chitinised; apex sharp, distal third with numerous sharp denticles. G2 (Fig. 7E) less than one-third G1 length, relatively straight, apex spatuliform. Male genital opening (gonopore) coxal; penis long. Female characters not known.

##### Remarks.

The type species of *Nectopanope* has been somewhat confused. Only one species, *Nectopanopelongipes*, was recognised in Anonymous (1891: 56) but both these names are nomina nuda. Wood-Mason (in Wood-Mason and Alcock 1891: 261, 262) provided valid descriptions for the genus and species, and included *N.rhodobaphes* as a second species. Ng et al. (2008: 80) noted that the type species of *Nectopanope* was *N.rhodobaphes* by monotypy, but this is not correct. Although Wood-Mason (in Wood-Mason and Alcock 1891) did not explicitly state which was the type species for *Nectopanope* Wood-Mason in Wood-Mason & Alcock, 1891, they wrote “*Nectopanoperhodobaphes*, gen. et sp. n., Wood-Mason” (Wood-Mason in Wood-Mason and Alcock 1891: 261). Under Article 68.2.1 of the Code (ICZN 1999), this is sufficient to recognise it as the type species of the genus (see Huys et al. 2014: 27). Alcock (1898: 213) later commented that *Nectopanope* should be restricted to *N.rhodobaphes* and that “*Nectopanopelongipes*, which was provisionally referred to this genus by Wood-Mason, who had insufficient material for examination, turns out, now that numerous good specimens have been dredged by the ‘Investigator,’ to be a Catometope belonging to the genus *Carcinoplax*.” Alcock (1899a: 64) repeated the same comments in his treatment of the deep-sea Crustacea of the Indian Seas. *Nectopanopelongipes* Wood-Mason in Wood-Mason & Alcock, 1891, is now generally regarded as a valid species in *Carcinoplax* H. Milne Edwards, 1853 (Goneplacidae MacLeay, 1838) (see Castro 2007).

*Nectopanope* Wood-Mason in Wood-Mason & Alcock, 1891, was originally placed in Cancridae Latreille, 1802, by Wood-Mason (in Wood-Mason and Alcock 1891) with Alcock (1898, 1899a) later transferring the genus to Xanthidae s. lato. Alcock (1898) recognized a new group in his xanthid subfamily Pilumninae, HeteropanopioidaAlcock, 1898, in which he included *Heteropanope* Stimpson, 1858, *Eurycarcinus* A. Milne-Edwards, 1867, and *Nectopanope*. Ng et al. (2008: 204) transferred *Nectopanope* to Xanthinae (Xanthidae) without explanation. This was necessary as *Heteropanope* and *Eurycarcinus* had already been moved to the Pilumnidae (present Pilumnoidea) by then (see Ng et al. 2018).

**Figure 2.**
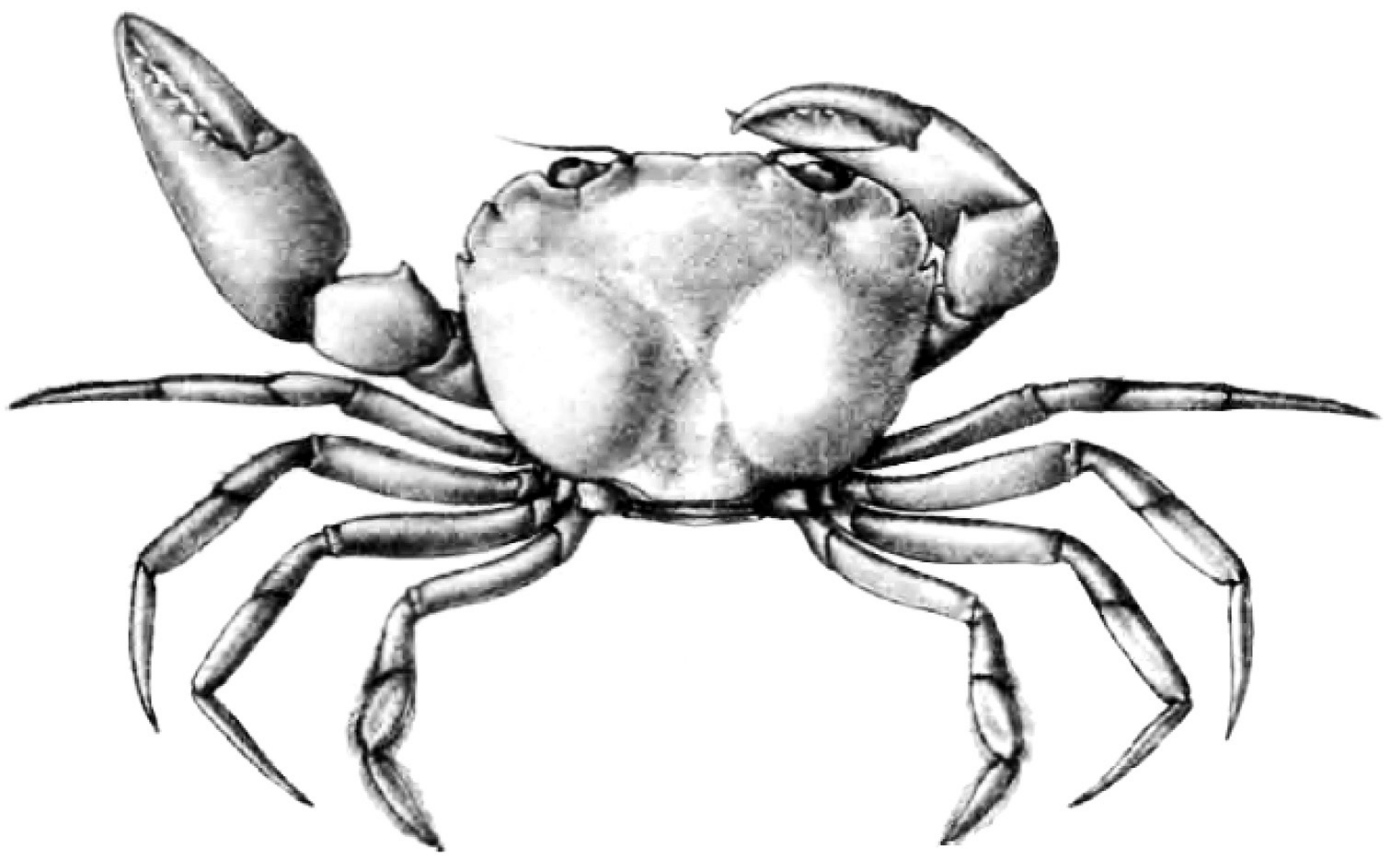
*Nectopanoperhodobaphes* Wood-Mason in Wood-Mason & Alcock, 1891 (after Alcock 1899a: pl. 38 fig. 6).

The family position of *Nectopanope* is difficult because its only species, *N.rhodobaphes*, has previously only been known from one female specimen. Wood-Mason (in Wood-Mason and Alcock 1891: 262) noted that he had “one specimen obtained at Station 96, 98 to 102 fathoms; the length of its carapace is 21.4 millim., and the greatest breadth between the points of the third teeth 29 millim.” Station 96 was in the Bay of Bengal, 18°30'N, 84°46'E, substrate is sand at a depth of 98–102 fathoms, and dated 4 March 1890 (Anonymous 1914). The sex of the specimen was not stated. Alcock (1899a: pl. 38 fig. 6) figured the specimen but it is not clear what its sex was (Fig. 2). Alcock (1898: 213; 1899a: 65) noted that they only had one female collected from the Godávari coast (in the Bay of Bengal) from 98–102 fathoms, that is the type. A search in the Zoological Survey of India in Calcutta for this specimen was not successful and it could not be located (S. Mitra, personal communication).

The study of the present male specimen resolves the systematic position of *Nectopanope*. The structures of the male pleon and gonopods leave no doubt that *Nectopanope* is in fact a member of Euryplacidae Stimpson, 1871. *Nectopanope* is only superficially resembles *Eurycarcinus* (and the Pilumnidae) due to similar carapace features. Their male pleons and gonopods, however, are completely different (cf. Ng et al. 2018).

**Figure 3.**
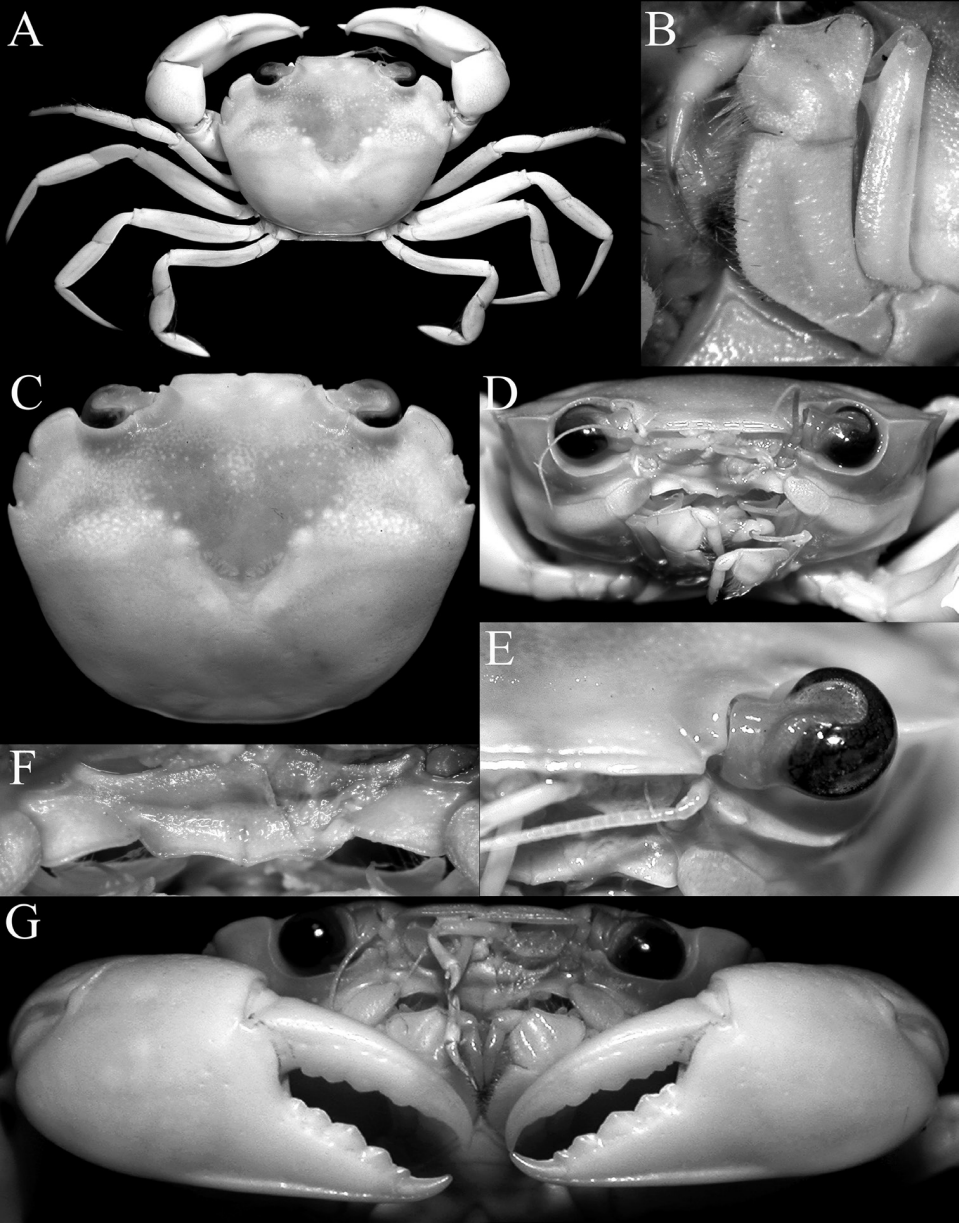
*Nectopanoperhodobaphes* Wood-Mason in Wood-Mason & Alcock, 1891, male (18.4 × 14.7 mm), India. **A** overall dorsal habitus **B** left third maxilliped **C** dorsal view of carapace **D** frontal view of cephalothorax **E** closeup of eye **F** epistome **G** outer view of chelae.

In Euryplacidae, the general shape and structure of the carapace as well as smoothness of the surfaces of *Nectopanope* most closely resembles *Psopheticoides* Sakai, 1969 (with only one species, *P. sanguineus* Sakai, 1969), from the western Pacific. They also share a similar eye morphology. The eye of *Psopheticoides* is large and is distinctly flattened dorsoventrally, with the structure appearing reniform (Castro and Ng 2010: fig. 36B). The eye of *Nectopanope* is relatively less distinctly flattened dorsoventrally and only weakly reniform in shape (Fig. 3D, E). No other euryplacids, however, have such a distinct eye structure which has been reported in other deep-sea brachyurans (e.g., *Hexaplax* Doflein, 1904, Hexapodidae; cf. Rahayu and Ng 2014).

**Figure 4.**
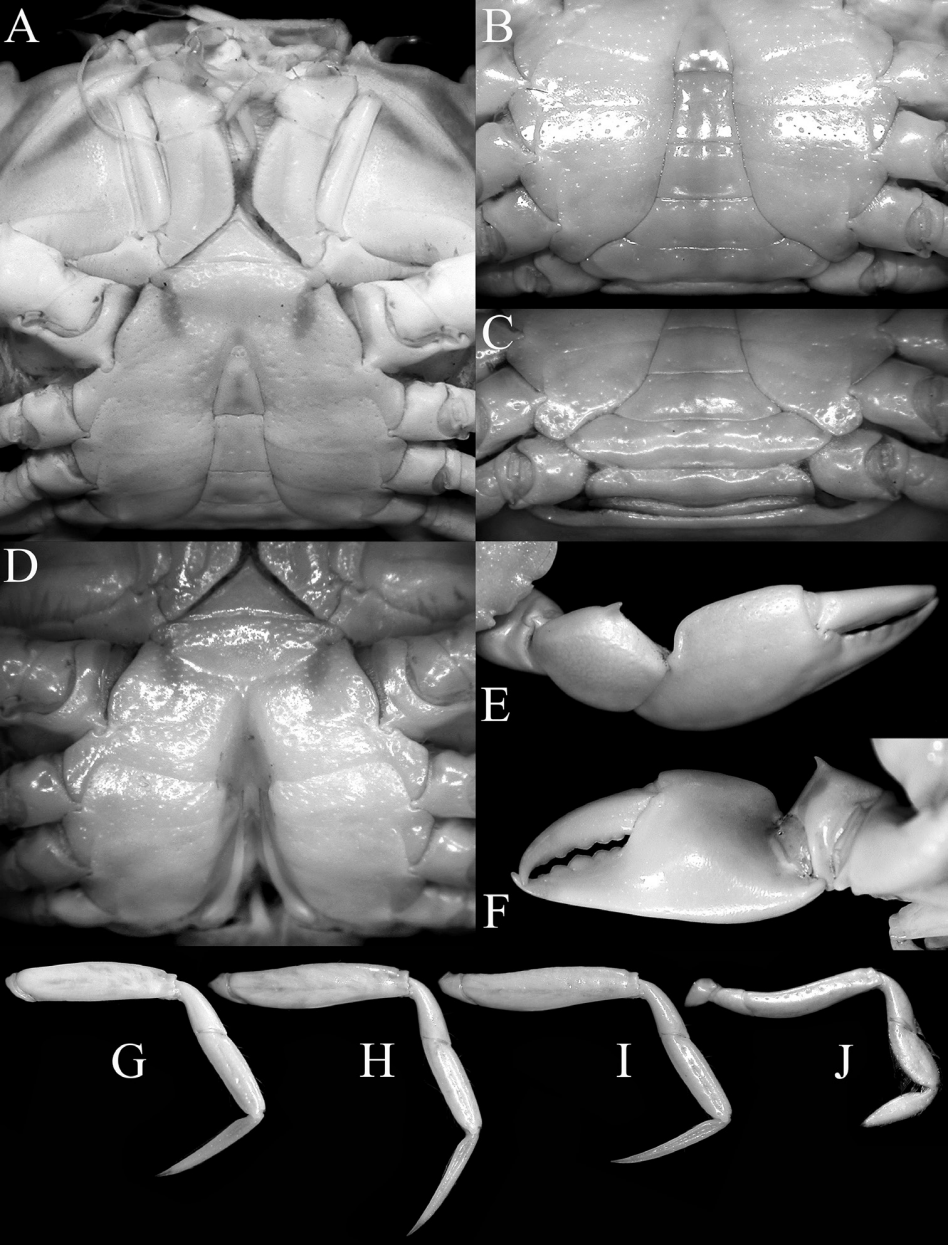
*Nectopanoperhodobaphes* Wood-Mason in Wood-Mason & Alcock, 1891, male (18.4 × 14.7 mm), India. **A** anterior thoracic sternum, pleon, buccal cavity and third maxillipeds **B** thoracic sternum and pleon **C** posterior thoracic sternum and pleon **D** anterior thoracic sternum and sternopleonal cavity **E** subdorsal view of right cheliped **F** inner view of right cheliped **G–J** second to fourth ambulatory legs, respectively (all to same scale).

The carapace anterolateral margin of *Nectopanope* has three distinct teeth (Figs 2, 3A, C) (with only two teeth in *Psopheticoides*, with the external orbital tooth very broad; Sakai 1969: text-fig. 16b; Sakai 1976: pl. 192, fig. 3). The external orbital tooth of *Psopheticoides*, however, is usually partially medially indented, and although the cleft is not deep, it gives the appearance of having three teeth on the anterolateral margin (cf. Sakai 1969: text-figs 16b, 18b; Sakai 1976: text-fig. 282a; Castro and Ng 2010: fig. 36A). The frontal margin in *Nectopanope* is medially indented (Figs 2, 3A, C) (margin entire in *Psopheticoides*; cf. Castro and Ng 2010: fig. 36A); the ischium of the third maxilliped is proportionately longer with the auriculiform anteroexternal angle of the merus more developed (Fig. 3B) (ischium of third maxilliped relatively shorter and the anterexternal angle of the merus less produced in *Psopheticoides*; cf. Castro and Ng 2010: fig. 36C); the cornea is weakly reniform (Fig. 3D, E) (cornea prominently reniform in *Psopheticoides*; cf. Castro and Ng 2010: fig. 36B); the male telson is proportionately shorter (Fig. 4A) (elongated and linguiform in *Psopheticoides*; cf. Castro and Ng 2010: fig. 36E); and the G1 is relatively straighter with the tip tapered to a tip (Fig. 7A–D) (G1 slightly sinuous with the tip arrow-shaped in *Psopheticoides*; cf. Castro and Ng 2010: fig. 38D, E).

#### Nectopanope rhodobaphes

Taxon classificationAnimaliaDecapodaEuryplacidae

Wood-Mason in Wood-Mason & Alcock, 1891

Figs 2 , 3 , 4 , 7A–E

Nectopanope rhodobaphes Wood-Mason in Wood-Mason & Alcock, 1891: 261; Alcock 1899a: pl. 38 fig. 6; Ng et al. 2008: 204 (list); Huys et al. 2014: 15, 27 (discussion).

##### Material examined.

1 male (18.4 × 14.7 mm), 7°27.978'N, 77°32.297'E, 200 m.

##### Diagnosis.

As for genus.

##### Description.

Carapace (Figs 2, 3A, C) transversely subhexagonal, 1.25 times wider than long; dorsal surface distinctly convex, smooth, without setae or granules; regions poorly defined, epigastric region not indicated, gastro-cardiac groove shallow. Front (Fig. 3A, C) lamellar, almost straight, smooth, with shallow median notch; postorbital region smooth, without trace of crest; front separated from supraorbital margin by small but distinct right-angled notch; lateral lobe triangular, small. Anterolateral margin (Fig. 3A, C) convex with three teeth including external orbital angle; external orbital angle broadly triangular, subtruncate; first lateral tooth triangular, tip directed anteriorly, separated from other teeth by deep-V-shaped notch, margin gently convex and entire to uneven; second lateral tooth acutely triangular, directed obliquely laterally. Posterolateral margin (Fig. 3A, C) gently convex, converging gradually towards gently convex posterior carapace margin. Suborbital, subhepatic, anterior half of pterygostomial regions (Fig. 3D) smooth. Orbits (Fig. 3D, E) wide, spherical, deep; supraorbital margin concave, smooth with distinct submedian fissure, gradually merging with external orbital tooth; suborbital tooth lined with small granules, with broad low tooth on inner edge, adjacent to antenna. Eye peduncles short, stout, with large subreniform (dorsoventrally flattened) cornea (Fig. 36D, E). Basal antennal article (articles 2 and 3) (Fig. 3D, G) rectangular, longer than broad, mobile, completely closing orbital hiatus. Basal antennular article subrectangular; flagellum long, folding transversely. Epistome (Fig. 3D, F) longitudinally narrow; posterior margin of epistome with prominent subtruncate median projection, with distinct longitudinal fissure; lateral margin almost straight, separated from median part by fissure. Endostomial ridge distinct, long.

Third maxillipeds (Fig. 3B) almost completely closing buccal cavern when closed; merus subquadrate, anteroexternal margin strongly auriculiform; ischium subrectangular, elongated, with submedian oblique sulcus, inner margin serrated, lined with dense stiff setae; exopod stout with prominent subdistal triangular tooth on inner margin, flagellum long, extending past width of merus.

Chelipeds (P1) (Figs 3A, G, 4E, F) unequal, right chela slightly larger; fingers slender, as long as palm; dorsal margin of palm rounded; distal half of chela with ventral margin (including entire pollex) possessing distinct subventral longitudinal sulcus, forming subcristiform ventral margin; outer surface of palm smooth; inner surface smooth with gently convex median part, ventro-proximal part with low lobiform rounded projection; cutting edge of pollex of major chela with prominent large triangular teeth; dactylus gently curved with 2 shallow longitudinal median sulci on outer margin (upper one deeper), cutting edge with large triangular teeth; fingers of minor chela similar to those on major chela.

Ambulatory legs (P2–P5) (Figs 3A, 4G–J) moderately long, slender; P3 longest; P2–P5 merus subcylindrical, slightly flattened laterally, outer surface smooth, glabrous, ventral margin smooth, dorsal margin almost entire; P5 merus gently up-curved; P2–P5 carpus short, outer surface glabrous, dorsal margin smooth; P2–P4 propodus of long, laterally flattened, with distinct shallow longitudinal median sulcus, lateral margins of distal third almost completely glabrous; P5 propodus ovate, laterally flattened, with distinct setae lining margins which partially obscure margin; P2–P4 dactylus elongated, falciform, smooth; P5 shortest, subspatuliform, margins lined with short setae; dactylo-propodal lock not distinct.

Thoracic sternum (Figs 4A, D) relatively wide, surface relatively smooth but with shallow uneven pits; sternites 1, 2 completely fused, distinctly triangular, lateral margins gently concave, separated from sternite 3 by distinct gently convex suture (towards buccal cavity); sternites 3, 4 fused with only lateral part of suture clearly visible, median part indicated by barely discernible shallow broad groove; sutures 4/5, 5/6, 7/8 medially interrupted, suture 6/7 almost complete, separated by very narrow gap; distinct median longitudinal groove extending across sternites 7, 8. Posterior edge of episternite 7 partially overlapping anterior part of P5 coxa and partially covering anterolateral part of pleonal somite 3 when closed. Sternopleonal cavity (Fig. 4A, D) deep, reaching nearly to anterior edge of sternite 4, just before sternite 3, defined by imaginary line connecting midpoint of coxae of chelipeds; pleon (Fig. 4C) completely covering sternite 8 when closed. Press-button of male pleonal locking mechanism (Fig. 4D) present as short spur-like structure on anterior quarter of sternite 5, just adjacent to sternite 4. Opening for penis coxal, penis relatively short, tubular, exiting at anterior edge of condyle of P5 coxa.

Pleon (Fig. 4A–C) narrow, slender, distinctly T-shaped; somites 3–6 trapezoidal, abruptly narrowing from somite 3–6; telson acutely triangular with convex lateral margins; somite 3 wide, reaching inner margins of P5 coxae; no part of thoracic sternite 8 exposed by closed pleon; somite 2 transversely shorter than somite 3 but reaching P5; somite 1 conspicuous, narrow, almost as wide as somite 2.

G1 (Fig. 7A–D) long, slender, almost straight; distal quarter distinctly chitinised, stiff; apex sharp, distal third with numerous sharp denticles, longer in some specimens than in others. G2 (Fig. 7E) less than one-third G1 length, relatively straight, apex spatuliform.

##### Remarks.

The colour of the fresh type specimen was described as “a beautiful deep-sea pink, with a dotted, V-shaped, white mark between the gastric and branchial regions.” (Wood-Mason in Wood-Mason and Alcock 1891: 262). The present preserved specimen, although faded, retains enough colour to suggest that in life, it had the colour and pattern described in the original description. This colour is somewhat similar to that known for *Psopheticoidessanguineus* which is red to pinkish-red all over but with a white ring on the median dorsal surface (Sakai 1976: pl. 192, fig. 3; Castro and Ng 2010: fig. 39C).

The type female (Fig. 2) shows the branchial regions distinctly swollen but this is probably due to parasites, although the specimen was not dissected. This has precedence in the Australian euryplacid *Eucratesexdentata* Haswell, 1882, in which one specimen has both sides of the branchial regions swollen and infected by bopyrids (cf. Castro and Ng 2010: fig. 10E).

#### *Henicoplax* Castro & Ng, 2010

##### Henicoplax maldivensis

Taxon classificationAnimaliaDecapodaEuryplacidae

(Rathbun, 1902)

Figs 5 , 7F–H

Goneplax maldivensis Rathbun, 1902: 124, figs 3–5; Guinot 1969: 518; Castro 2007: 686, 687 [list].
“?[Goneplax] maldivensis”: Guinot 1971: 1081 [list].
Otmaroplax maldivensis : Števčić 2005: 134 [genus name nomen nudum]
“Heteroplax” maldivensis: Ng et al. 2008: 78, 79 [in list].
Henicoplax maldivensis : Castro and Ng 2010: 61, figs 22A–E, 24D–F.

###### Material examined.

1 male (9.9 × 6.3 mm), 7°27.978'N, 77°32.297'E, 100 m.

###### Remarks.

*Henicoplax* Castro & Ng, 2010, was established for Indo-West Pacific species that had been previously classified in *Goneplax* Leach, 1814, or *Heteroplax*Stimpson, 1858. Five species are currently recognised: *H.eriochir* Castro & Ng, 2010, *H.maldivensis* (Rathbun, 1902) [type species], *H.nitida* (Miers, 1879a), *H.pilimeles* Castro & Ng, 2010, and *H.trachydactylus* Castro & Ng, 2010.

**Figure 5.**
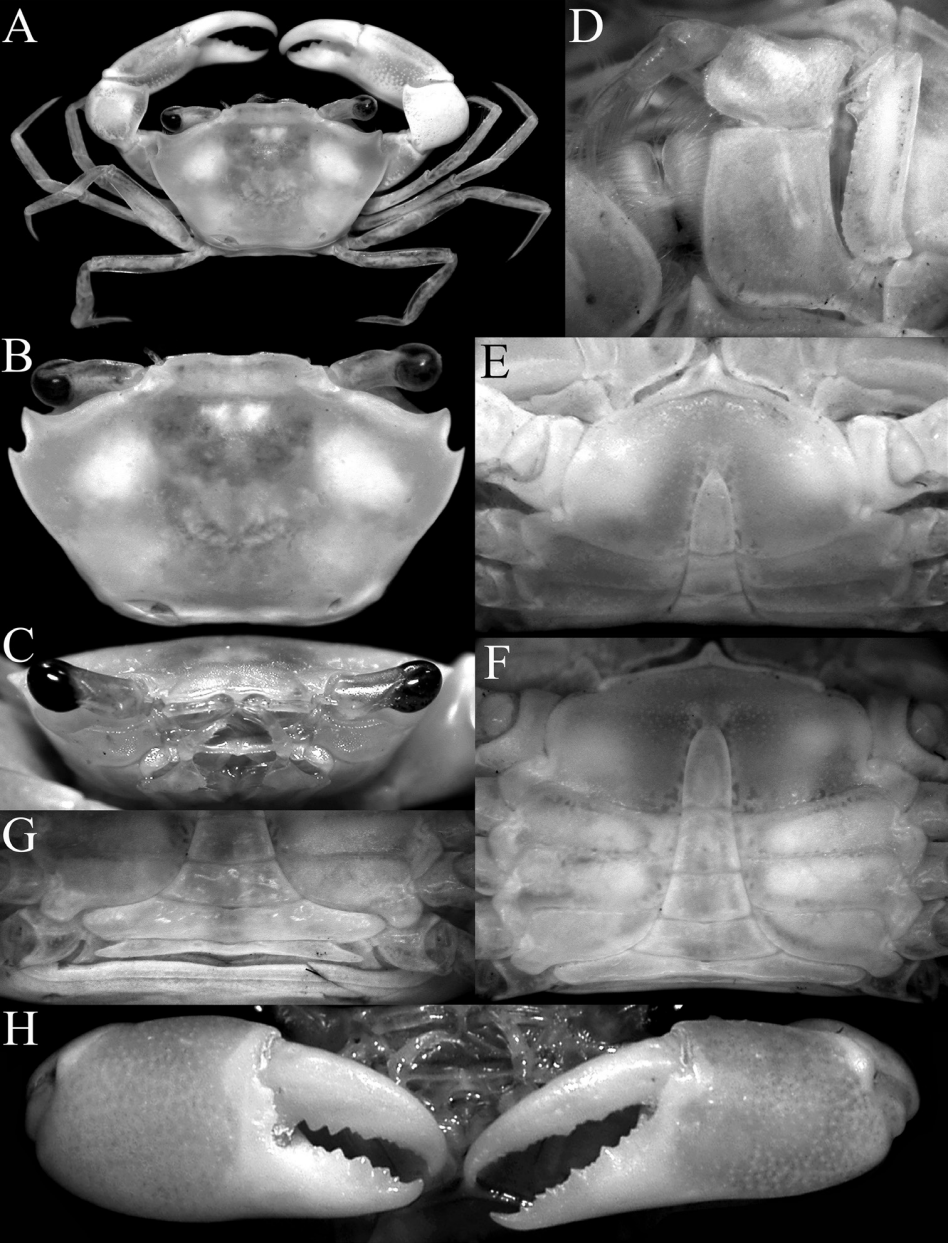
*Henicoplaxmaldivensis* (Rathbun, 1902), male (9.9 × 6.3 mm), India. **A** overall dorsal habitus **B** dorsal view of carapace **C** frontal view of cephalothorax **D** left third maxilliped **E** anterior thoracic sternum and pleon **F** thoracic sternum and pleon **G** posterior thoracic sternum and pleon **H** outer view of chelae.

The present specimen is clearly *H.maldivensis* as redescribed and figured at length by Castro and Ng (2010: 61). The species was previously known only from the holotype male (7.4 × 4.8 mm) obtained from Gan Island in Addu Atoll in the Maldives. The present male differs from the type male in having the frontal margin slightly more sinuous and the cleft between the external orbital tooth and the anterolateral tooth is more U-shaped (Fig. 5A, B) (versus frontal margin almost straight and the lateral carapace cleft being V-shaped in the holotype; cf. Castro and Ng 2010: fig. 22A); and while the G1 shape is similar, the distal spination is relatively less pronounced and the tip is sharply tapering (Fig. 7F, G) (versus distal half with relatively more spines and the tip is subtruncate in the holotype; cf. Castro and Ng 2010: fig. 24D, E). The differences are not substantial and can easily be explained by variation and size, the present male (9.9 × 6.3 mm) being larger than the type (7.4 × 4.8 mm).

Castro and Ng (2010) showed that records of “*H.nitida*” from the Andaman Sea should be referred to a new species, *H.pilimenes*; and indicated that true *H.nitida* should be restricted to East Asia. The records of “*H.nitida*” from the Gulf of Martaban (south of Myanmar) and off Madras in India by Henderson (1893: 379) are almost certainly not this species. The Myanmar material is probably *H.pilimenes*, while that from India may be this species or even *H.maldivensis* instead. Specimens will need to be re-examined to be certain.

*Heteroplaxmaldivensis* is thus far known for certain only from the Maldives (Rathbun 1902; Castro and Ng 2010) and the present specimen represents the first confirmed presence of this species in India.

### Family Parthenopidae Macleay, 1838

#### *Pseudolambrus* Paulson, 1875

##### Pseudolambrus beaumonti

Taxon classificationAnimaliaDecapodaParthenopidae

(Alcock, 1895)

Fig. 6A

###### Material examined.

1 female (9.4 × 8.8 mm), 7°48.004'N, 77°27.754'E, 50 m.

###### Remarks.

This species was described from off Sri Lanka and Andamans by Alcock (1895) and has since been reported from Mauritius and Japan (Flipse 1930; Michel 1964; Sakai 1976). Ng and Rahayu (2010) figured the syntypes of the species.

**Figure 6.**
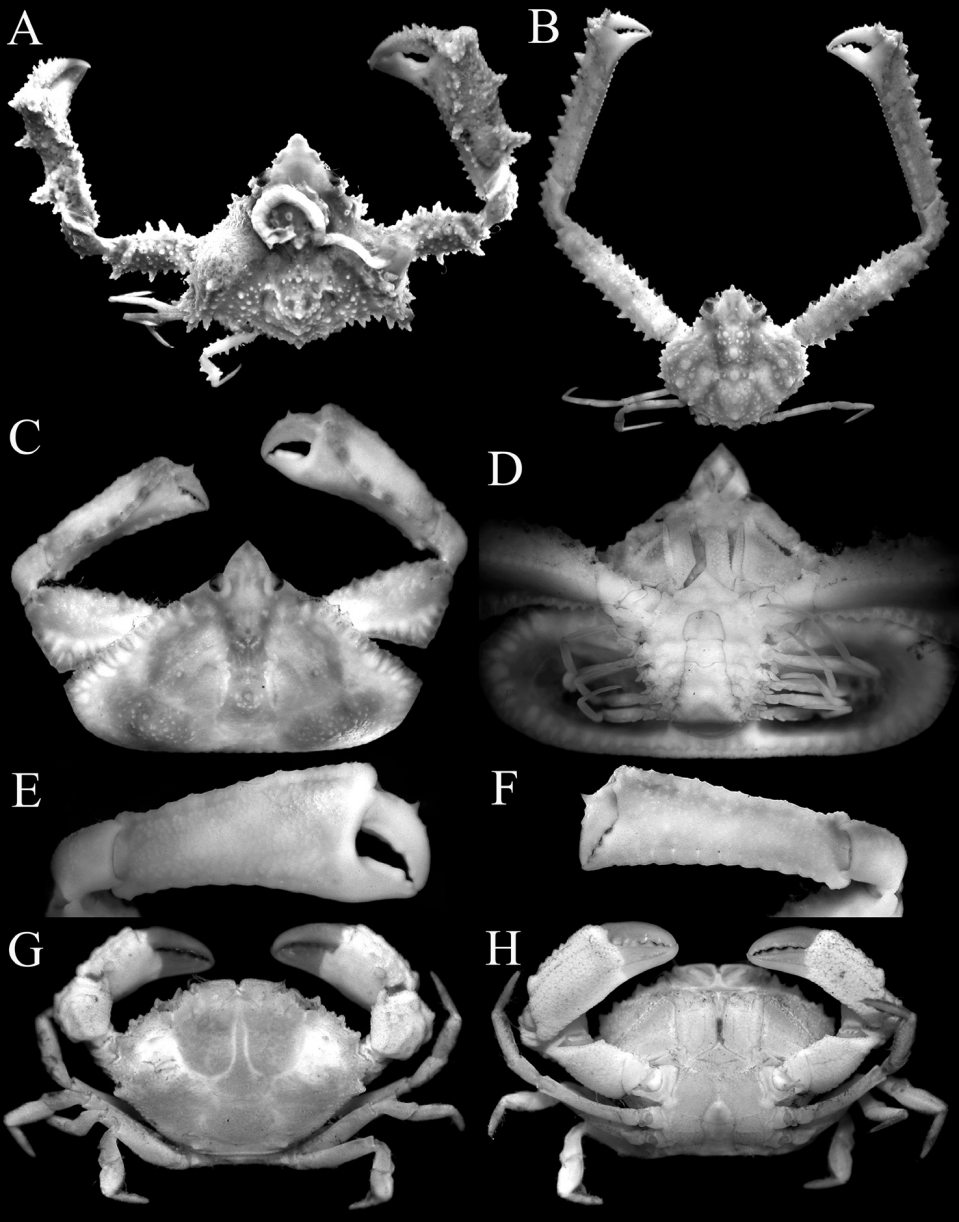
**A***Pseudolambrusbeaumonti* (Alcock, 1895), female (9.4 × 8.8 mm) **B***Rhinolambruscontrarius* (Herbst, 1804), female (10.5 × 10.0 mm) **C–F***Cryptopodiacollifer* Flipse, 1930, male (17.5 × 10.6 mm) **G, H***Paraxanthodescumatodes* (MacGilchrist, 1905), male (8.3 × 5.5 mm). **A, B, C, G, H** overall dorsal habitus **D** ventral view of cephalothorax **E, F** outer view of chelae.

#### *Rhinolambrus* A. Milne-Edwards, 1878

##### Rhinolambrus contrarius

Taxon classificationAnimaliaDecapodaParthenopidae

(Herbst, 1804)

Fig. 6B

###### Material examined.

1 young female (10.5 × 10.0 mm), 3 juveniles (largest 6.8 × 6.8 mm), 7°48.004'N, 77°27.754'E, 50 m.

###### Remarks.

Herbst (1804: 9) described this species from material from somewhere in the “East Indies” and as far as is known, the type is lost (Sakai 1999). This is the type species of *Rhinolambrus* A. Milne-Edwards, 1878. The species has a wide Indo-West Pacific distribution (see Flipse 1930; Sakai 1976); and in India has been reported from various parts of Tamil Nadu and Andamans (Henderson 1893; Jeyabaskaran et al. 2000; Kathirvel and Gokul 2010; Dev Roy and Nandi 2012; Vidhya et al. 2017).

The present materials are all juveniles, with none of the gonopod structures of the males developed even though the chelipeds are elongated. The pronounced “neck-like” constriction in adults of this species has still not developed (Fig. 6B).

#### *Cryptopodia* H. Milne Edwards, 1834

##### Cryptopodia collifer

Taxon classificationAnimaliaDecapodaParthenopidae

Flipse, 1930

Figs 6D–F , 7I–M

Cryptopodia collifer Flipse, 1930: 66, fig. 41; Serène 1968: 62 (list); Shen et al. 1982: 144, pl. 1 fig. 8; Dai et al. 1986: 160, pl. 21 fig. 8, text-fig. 91; Dai and Yang 1991: 176, pl. 21 fig. 8, text-fig. 91; Cai et al. 1994: 584 (list); Chiong and Ng 1998: 189, fig. 22; Davie et al. 2002: 322 (list); Ng and Davie 2002: 372 (list); Ng et al. 2008: 130 (list).

###### Material examined.

1 male (17.5 × 10.6 mm), 7°27.978'N, 77°32.297'E, 100 m.

###### Remarks.

Five species of *Cryptopodia* H. Milne Edwards, 1834, are known from India (Trivedi et al. 2018): *C.angulata* H. Milne Edwards & Lucas, 1841, *C.echinosa* Chiong & Ng, 1998, *C.fornicata* (Fabricius, 1787), *C.patula* Chiong & Ng, 1998, and *C.spatulifrons* Miers, 1879b. The addition of *C.collifer* Flipse, 1930, not previously known from the Indian Ocean, is notable. *Cryptopodiacollifer* Flipse, 1930, was described from a single female specimen from eastern Indonesia and has since been reported from China (Shen et al. 1982). In an unpublished study, S.H. Tan and the first author examined specimen of this species from off Phuket, Philippines and Fiji, including males, and they agree well with the specimen obtained here from India, and as figured by Chiong and Ng (1998: fig. 22).

The lateral margins of the rostrum are straight in the holotype of *C.collifer* (cf. Chiong and Ng 1998: fig. 22A) but are gently convex in the present male (Fig. 6C), as was figured by Shen et al. (1982: pl. 1 fig. 8) for the Chinese specimen. The male telson of *C.collifer* is semi-circular in shape (Fig. 6D), and is distinct from the more triangular shapes of other *Cryptopodia* species (see Chiong and Ng 1998). The G1 structure of *C.collifer* is most similar to that of *C.pan* Laurie, 1906, from the Indo-West Pacific (cf. Chiong and Ng 1998: fig. 24A, B, D–K), but the latter species is easily distinguished by its third maxilliped being distinctly swollen (Chiong and Ng 1998: fig. 23C). The third maxilliped of *C.collifer*, like those of other congeners, is quadrate and not inflated (Fig. 6D).

### Family Xanthidae Macleay, 1838

#### *Paraxanthodes* Guinot, 1968

##### Paraxanthodes cumatodes

Taxon classificationAnimaliaDecapodaXanthidae

(MacGilchrist, 1905)

Figs 6G, H , 7N–P

Xanthodes cumatodes MacGilchrist, 1905: 205; Alcock et al. 1907: pl. 79 fig. 1, 1A.
Xanthias cumatodes : Balss 1929: 24; Stephensen 1946: 148.
Paraxanthodes cumatodes : Guinot 1968: 723, fig. 60; Guinot 1971: 1069; Serène 1968: 77; Serène 1984: 209, pl. 30 fig. C; Ng et al. 2008: 204 (list); Mendoza et al. 2012: 3, fig. 1D–F, 2E–I.

###### Material examined.

1 male (8.3 × 5.5 mm), 8°58.270'N, 76°17.365'E, 50 m.

###### Remarks.

The taxonomic problems associated with *Paraxanthodes* Guinot, 1968, and the allied genera *Monodaeus* Guinot, 1967, and *Medaeops* Guinot, 1967, and *Takedax* Mendoza & Ng, 2012, as well the generic affinities of species previously classified in these taxa have been discussed at length by Lai et al. (2011) and Mendoza and Ng (2012). While the genera are distinct at the genetic level, the available morphological characters are not always reliable; and work is still ongoing to clarify their affinities. Mendoza et al. (2012) discussed the generic position of *P.cumatodes*, and suggested that it may not be congeneric with *P.obtusidens* (Sakai, 1965), the type species of *Paraxanthodes*.

**Figure 7.**
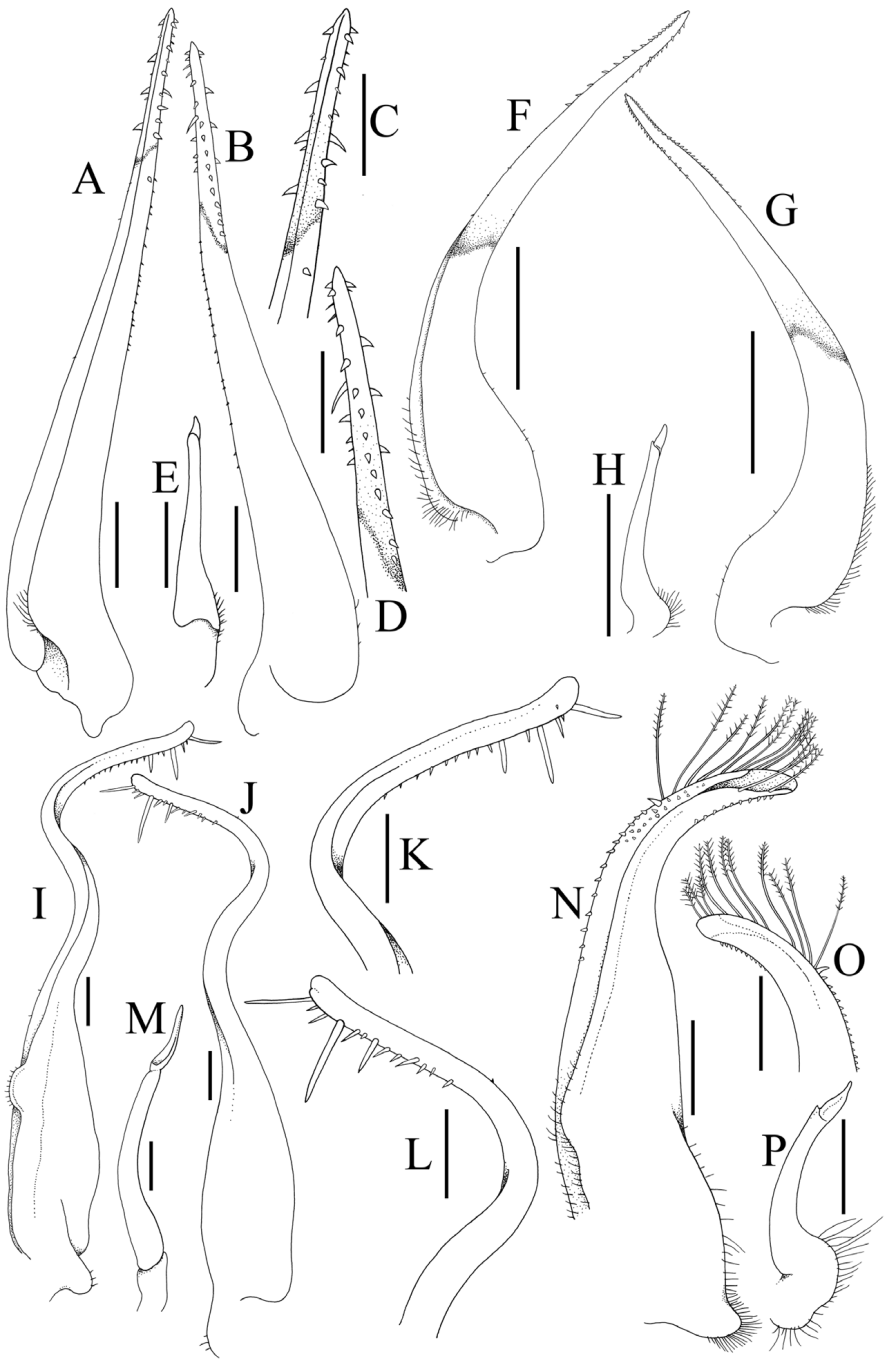
Gonopods. **A–E***Nectopanoperhodobaphes* Wood-Mason in Wood-Mason & Alcock, 1891, male (18.4 × 14.7 mm) **F–H***Henicoplaxmaldivensis* (Rathbun, 1902), male (9.9 × 6.3 mm) **I–M***Cryptopodiacollifer* Flipse, 1930, male (17.5 × 10.6 mm) **N–P***Paraxanthodescumatodes* (MacGilchrist, 1905), male (8.3 × 5.5 mm). **A, F** left G1 (ventral view) **B, G** left G1 (dorsal view) **C** distal part of left G1 (ventral view) **D** distal part of left G1 (dorsal view); left G2. Scales bars: 0.5 mm (**A, B, E–H–P**); 0.25 mm (**C, D**).

We refer the present specimen to *P.cumatodes*, described from the western Indian Ocean by MacGilchrist (1905), with doubt because of its relatively small size. It differs from typical *P.cumatodes* (see Alcock and Annandale 1907: pl. 79, fig. 1, 1A; Guinot 1968: fig. 60; Serène 1984: pl. 30C; Mendoza et al. 2012: fig. 1D–F) in having the carapace proportionately less broad, the grooves and ridges on the dorsal carapace surface less prominent and the grooves on the thoracic sternum relatively shallower (Fig. 6G, H). The G1 of the present specimen is developed and its structure agrees relatively well with that figured by Mendoza et al. (2012: fig. 2E, G–I) for *P.cumatodes*, except that the distal half is more gently curved and the distal setae less dense (Fig. 7N, O).

## Supplementary Material

XML Treatment for Notosceles serratifrons

XML Treatment for Nursilia tonsor

XML Treatment for Arcania gracilis

XML Treatment for Coleusia urania

XML Treatment for Xiphonectes tuberculosus

XML Treatment for Monomia argentata argentata

XML Treatment for Thalamita macrodonta

XML Treatment for Nectopanope

XML Treatment for Nectopanope rhodobaphes

XML Treatment for Henicoplax maldivensis

XML Treatment for Pseudolambrus beaumonti

XML Treatment for Rhinolambrus contrarius

XML Treatment for Cryptopodia collifer

XML Treatment for Paraxanthodes cumatodes
